# Discovery of a Secretory Granule Lumen-Enriched Serum Protein Signature in Resectable Pancreatic Ductal Adenocarcinoma

**DOI:** 10.3390/medicina62030605

**Published:** 2026-03-23

**Authors:** Septimiu Alex Moldovan, Maria Iacobescu, Emil Ioan Moiș, Florin Graur, Luminiţa Furcea, Florin Zaharie, Andra Ciocan, Maria-Andreea Soporan, Ioana-Ecaterina Pralea, Simona Mirel, Mihaela Ştefana Moldovan, Andrada Seicean, Vlad Ionuț Nechita, Cristina Adela Iuga, Nadim Al Hajjar

**Affiliations:** 1 Department of Surgery, “Octavian Fodor” Regional Institute of Gastroenterology and Hepatology, Croitorilor Str., No. 19–21, 400162 Cluj-Napoca, Romaniadrmoisemil@gmail.com (E.I.M.); graurf@yahoo.com (F.G.); nechita.vlad@umfcluj.ro (V.I.N.);; 2 Department of Surgery, “Iuliu Hațieganu” University of Medicine and Pharmacy, Croitorilor Str., No. 19–21, 400162 Cluj-Napoca, Romania; 3 Department of Personalized Medicine and Rare Diseases, MedFuture—Institute for Biomedical Research, “Iuliu Hațieganu” University of Medicine and Pharmacy, Louis Pasteur Str., No. 4, 400347 Cluj-Napoca, Romania; 4 Department of Pharmaceutical Analysis, Faculty of Pharmacy, “Iuliu Hațieganu” University of Medicine and Pharmacy, Louis Pasteur Str., No. 6, 400347 Cluj-Napoca, Romania; 5 Department of Medical Devices, “Iuliu Hațieganu” University of Medicine and Pharmacy, Louis Pasteur Str., No. 4, 400349 Cluj-Napoca, Romania; 6 Department of Endocrinology, County Emergency Hospital, Endocrinology Clinic, Louis Pasteur Str., No. 3–5, 400349 Cluj-Napoca, Romania; 7 Department of Gastroenterology, “Octavian Fodor” Regional Institute of Gastroenterology and Hepatology, Croitorilor Str., No. 19–21, 400162 Cluj-Napoca, Romania; 8 Department of Gastroenterology, “Iuliu Hațieganu” University of Medicine and Pharmacy, Croitorilor Str., No. 19–21, 400162 Cluj-Napoca, Romania; 9 Department of Medical Informatics and Biostatistics, “Iuliu Hațieganu” University of Medicine and Pharmacy, Louis Pasteur Str., No. 6, 400349 Cluj-Napoca, Romania

**Keywords:** pancreatic ductal adenocarcinoma, resectable pancreatic cancer, serum proteomics, quantitative proteomics, circulating biomarkers, multimarker panel, diagnostic performance, liquid biopsy

## Abstract

*Background and Objectives*: Serum biomarker discovery in resectable pancreatic ductal adenocarcinoma (PDAC) remains a critical unmet need, as over 80% of patients present with unresectable disease. Serum proteomics offers a promising approach for identifying circulating biomarkers associated with early-stage disease; however, clinical translation has been limited by inconsistent validation and the absence of clinically relevant comparator populations. *Materials and Methods*: We performed a discovery-phase study using data-independent acquisition mass spectrometry-based serum proteomics in 35 patients with resectable, non-metastatic PDAC and 34 non-cancer controls without hepato-biliary-pancreatic disease. Following quality filtering (≥80% detection threshold), 407 proteins were retained for analysis. Differential abundance was assessed using Welch’s *t*-test with Benjamini–Hochberg correction (FDR < 0.01, |FC| ≥ 1.5). Diagnostic performance was evaluated using receiver operating characteristic (ROC) analysis and logistic regression with repeated stratified 5-fold cross-validation (100 repetitions) and bootstrap resampling (1000 iterations). Functional enrichment analysis was performed using g:Profiler. *Results*: Ninety proteins were significantly altered in PDAC (50 increased, 40 decreased). Inter-alpha-trypsin inhibitor heavy chain H3 (ITIH3) demonstrated the highest individual diagnostic performance (AUC = 0.90), followed by coagulation factor XIII A chain (F13A1; AUC = 0.89) and ferritin light chain (FTL; AUC = 0.86). Functional enrichment revealed significant overrepresentation of secretory granule lumen components (adjusted *p* = 0.001) and complement/coagulation pathways (adjusted *p* < 0.001). An enrichment-guided three-protein panel (ITIH3, F13A1, and FTL) achieved an AUC of 0.98 (95% CI: 0.95–1.00), with a cross-validated mean AUC of 0.96, sensitivity of 83% (95% CI: 66.4–93.4%), and specificity of 100% (95% CI: 89.7–100%) within the discovery cohort. *Conclusions*: This discovery-phase study identifies a biologically coherent serum protein signature enriched for secretory granule lumen components in resectable PDAC. The three-protein panel demonstrates strong internal validation performance; however, these estimates may be optimistic due to feature selection performed prior to cross-validation. External validation in independent cohorts—including chronic pancreatitis controls and parallel CA19-9 assessment—will be essential to determine clinical applicability.

## 1. Introduction

Pancreatic ductal adenocarcinoma (PDAC) survival has improved only marginally over four decades—from approximately 5% to 12–15%—a trajectory starkly different from other solid tumors where early detection or discrimination strategies have transformed outcomes [1,2]. This poor prognosis is largely attributable to late-stage diagnosis: over 80% of patients present with locally advanced or metastatic disease when curative resection is no longer feasible [3,4]. Notably, patients with resectable stage Ia PDAC have a 5-year survival rate of approximately 84%, underscoring the transformative potential of early detection [5].

Pancreaticoduodenectomy (Whipple procedure) remains the standard surgical treatment for PDAC of the pancreatic head and the only potentially curative option for resectable disease. However, only 15–20% of patients have resectable disease at diagnosis [6,7]. Even among patients eligible for curative-intent resection, long-term survival remains limited due to high recurrence rates and aggressive tumor biology. Total pancreatectomy is reserved for situations where partial resection cannot guarantee oncological radicality, such as multifocal disease, extensive ductal involvement, or severe gland fibrosis.

Carbohydrate antigen 19-9 (CA19-9) is currently the only biomarker approved for clinical use in PDAC. However, CA19-9 achieves only 70–80% sensitivity and 80–90% specificity overall, with performance deteriorating substantially in early-stage disease—sensitivity falls to approximately 50–55% for stage I tumors [8,9,10]. Furthermore, CA19-9 elevations occur in 10–30% of patients with chronic pancreatitis and obstructive jaundice, precisely the clinical scenarios where diagnostic discrimination is most needed [11]. Approximately 5–10% of patients are Lewis antigen-negative non-secretors who do not express CA19-9. These limitations underscore the urgent need for novel, noninvasive biomarkers capable of improving discrimination of resectable PDAC from non-cancer patients.

Recent developments in liquid biopsy technologies—including circulating tumor DNA, exosome-based signatures, and multiplex blood biomarker panels—have shown promise for non-invasive detection of PDAC [12]. Recent studies demonstrate that multi-protein signatures combining CA19-9 with markers such as Thrombospondin 2 (THBS2), Tissue inhibitor of metalloproteinases 1 (TIMP1), Leucine-rich alpha-2-glycoprotein 1 (LRG1) can achieve area under the receiver operating characteristic curve (AUC) values ranging from 0.92 to 0.97 for early-stage PDAC detection, substantially outperforming CA19-9 alone [13,14].

Serum proteomics represents a promising approach for PDAC biomarker discovery because the disease induces systemic alterations—including hypercoagulability, acute-phase responses, and metabolic changes—that can be detected in circulation and may reflect tumor–host interactions. Unlike genomic approaches that capture tumor-intrinsic mutations, proteomics captures the integrated tumor-host response, potentially identifying patients whose systemic biology has shifted toward malignancy even when tumor burden remains small. Advances in mass spectrometry-based technologies—including high-resolution mass analyzers, nanoscale liquid chromatography, and data-independent acquisition (DIA) approaches—have substantially enhanced proteome coverage, sensitivity, and quantitative reproducibility [15].

Extensive proteomic investigations have characterized PDAC-associated alterations across tumor tissue, pancreatic secretions, extracellular vesicles, and peripheral blood [16]. Integrative analyses consistently highlight dysregulation of pathways related to inflammation, extracellular matrix remodeling, immune modulation, and metabolic reprogramming—processes central to PDAC pathogenesis [17,18]. These biological processes are reflected in circulating proteins and extracellular vesicles, which mirror both tumor-intrinsic mechanisms and the host response to malignancy [19,20].

Peripheral blood represents an attractive source for biomarker discovery due to its minimal invasiveness and suitability for longitudinal monitoring. Recent plasma and serum proteomic investigations demonstrate that multi-protein signatures can outperform CA19-9 alone for diagnostic applications in PDAC [13,21]. However, translation into clinical practice has been limited by biological heterogeneity, technical variability, restricted cross-cohort validation, and inconsistent analytical pipelines. Moreover, many prior serum proteomic investigations have included advanced or metastatic PDAC cases, potentially confounding early disease-specific signatures [22,23,24,25,26,27].

We hypothesized that resectable, non-metastatic pancreatic ductal adenocarcinoma induces coordinated alterations in circulating proteins reflecting tumor–stroma interactions and systemic inflammatory responses, detectable through high-resolution serum proteomics. The primary objective of this discovery-phase study was to identify serum proteins capable of discriminating patients with resectable PDAC from non-cancer controls using data-independent acquisition mass spectrometry. Secondary exploratory objectives included: (i) evaluation of diagnostic performance of individual proteins using receiver operating characteristic (ROC) analysis; (ii) assessment of biologically coherent multimarker panels through functional enrichment analysis and logistic regression modeling; and (iii) investigation of associations between prioritized proteins and clinicopathologic characteristics within the PDAC cohort. The reported performance metrics should be interpreted as exploratory signals suitable for hypothesis generation, with independent validation in clinically relevant populations—including chronic pancreatitis and benign biliary disease—required to determine true diagnostic utility.

## 2. Materials and Methods

### 2.1. Study Design and Participants

This prospective, single-center biomarker discovery study was conducted at the “Octavian Fodor” Regional Institute of Gastroenterology and Hepatology, Cluj-Napoca, Romania, between 01 June 2023 and 31 December 2025. The study was approved by the Institutional Ethics Committee (approval no. 1125/30 January 2023) and the Ethics Committee of “Iuliu Hațieganu” University of Medicine and Pharmacy (approval no. AVZ 66/4 May 2023), and conducted in accordance with the Declaration of Helsinki. All participants provided written informed consent prior to enrollment.

PDAC cohort. Eligible patients had cephalic pancreatic tumors with either (i) non-diagnostic/inconclusive preoperative biopsy or (ii) histologically confirmed PDAC on preoperative biopsy. Only patients who underwent pancreatic resection with curative intent and had PDAC confirmed on final pathology were included. Exclusion criteria were: intraoperative findings of non-resectability (distant metastases, direct invasion of adjacent organs, or major vascular invasion); final pathology revealing non-PDAC malignancies (ampullary carcinoma, distal cholangiocarcinoma), premalignant lesions, or benign conditions (chronic pancreatitis).

Control cohort. Control participants were prospectively recruited from patients evaluated for non-malignant surgical conditions, most commonly abdominal wall or diaphragmatic hernias (inguinal, femoral, umbilical, or hiatal). Eligible individuals had no history of hepato-biliary-pancreatic disease, no prior or current malignancy, and no severe systemic conditions expected to substantially influence circulating protein profiles. The absence of hepato-biliary-pancreatic pathology was established through routine preoperative clinical evaluation, including medical history, physical examination, standard laboratory testing, and abdominal imaging when clinically indicated as part of the surgical assessment. Individuals with clinical, biochemical, or imaging findings suggestive of pancreatic, biliary, or hepatic disease were excluded from the study. Staging and pathological assessment. Tumor staging followed the UICC TNM Classification of Malignant Tumours, 8th edition. Pathological parameters recorded included T stage (pT2–pT3), N stage (pN0–pN2), metastasis status (pM0), lymphovascular invasion (L), vascular invasion (V), perineural invasion (Pn), tumor grade (G), and resection margin status (R0/R1).

The final study cohort comprised 35 patients with pancreatic ductal adenocarcinoma (PDAC) and 34 control subjects. Demographic and baseline clinical characteristics were summarized descriptively. Continuous variables are reported as mean ± standard deviation (SD) for normally distributed data and median with interquartile range (IQR) for non-normally distributed variables, while categorical variables are presented as counts and percentages (n, %). To assess potential baseline imbalance between cohorts, standardized mean differences (SMD) were calculated for key demographic variables, including age and sex.

The present study was designed as a discovery-phase proteomic investigation, and the sample size was determined primarily by feasibility considerations inherent to high-resolution mass spectrometry-based serum proteomics. These constraints include the substantial costs and technical demands of data-independent acquisition workflows, the requirement for stringent sample quality control, and the availability of patients meeting strict clinical inclusion criteria for resectable, non-metastatic PDAC with confirmed histopathology. We acknowledge that relatively small cohorts in omics-based discovery studies may increase the risk of effect-size inflation and model overfitting. To mitigate these risks, we applied stringent statistical thresholds, multiple testing correction using the Benjamini–Hochberg procedure, and internal validation strategies including repeated stratified cross-validation and bootstrap resampling, as detailed in the next sections. Nevertheless, the findings should be interpreted as hypothesis-generating, and independent validation in larger, multi-center cohorts will be required to confirm the diagnostic performance and generalizability of the identified biomarker panel.

Because this study used a case–control design, the reported diagnostic performance estimates may be higher than those observed in prospective screening settings, and should therefore be interpreted as exploratory.

### 2.2. Sample Collection and Processing

Peripheral venous blood was collected prior to any surgical or oncological intervention using standardized procedures. Blood was drawn into serum separator tubes and allowed to clot for 30 min at room temperature. Serum was obtained by centrifugation (2000× *g*, 10 min, 4 °C), aliquoted (500 µL) into protein low-binding tubes (Eppendorf LoBind, Hamburg, Germany), and stored at −80 °C until analysis.

### 2.3. Depletion of High-Abundance Proteins

Fourteen high-abundance serum proteins were depleted using multi-affinity chromatography (MARS14-Human, Agilent Technologies, Waldbronn, Germany; catalog no. 5188-6557) according to manufacturer instructions. Depleted proteins included: albumin, IgG, IgA, transferrin, haptoglobin, α1-antitrypsin, fibrinogen, α2-macroglobulin, α1-acid glycoprotein, IgM, apolipoprotein A-I, apolipoprotein A-II, complement C3, and transthyretin. Identical input volumes (25 µL serum) were used across all samples. Proteins were concentrated by trichloroacetic acid precipitation (15% final concentration), and pellets were resuspended in urea/thiourea buffer (8 M/2 M).

### 2.4. Proteolytic Digestion

Protein concentration was determined by Bradford microplate assay (Invitrogen, Waltham, MA, USA). For each sample, 4 µg protein underwent reduction (2.5 mM dithiothreitol, 30 min, 37 °C), alkylation (10 mM iodoacetamide, 15 min, 37 °C, protected from light), and overnight tryptic digestion (sequencing-grade trypsin, Merck KGaA, Darmstadt, Germany; 1:25 enzyme-to-protein ratio, 37 °C). Digestion was quenched with 1% acetic acid. Peptides were desalted using C18 ZipTips (Millipore-Sigma, Burlington, MA, USA), lyophilized, and reconstituted in 0.1% formic acid to a final concentration of 0.1 µg/µL. The non-human yeast alcohol dehydrogenase (ADH; accession P00330, Waters Corporation, Milford, MA, USA; catalog no. 186002328), spiked at a constant amount (75 fmol per sample), was used as an external reference to monitor instrument performance and data consistency across runs. The spike-in standard was not used to derive absolute protein concentrations but served as a quality control measure within the data-independent acquisition workflow. Protein abundance values reported in this study therefore represent normalized relative protein abundances derived from label-free quantification.

### 2.5. Nano-LC-HDMS^E^ Analysis

Nano-liquid chromatography–high-definition mass spectrometry (nano-LC-HDMS^E^) was performed as previously described. Briefly, 300 ng peptides were separated on an ACQUITY UPLC M-Class HSS T3 column (Waters Corporation) using a 120-min non-linear gradient (5–85% acetonitrile in 0.1% formic acid) at 300 nL/min. Mass spectrometry analysis was performed on a SYNAPT G2-Si HDMS instrument (Waters Corporation) in data-independent acquisition (DIA) mode with alternating low- and elevated-energy scans. Raw data were acquired using MassLynx™ (v1.74.2662). Detailed instrument parameters are provided in Supplementary Methods.

### 2.6. Database Search and Protein Identification

Raw data were processed using Progenesis QI for Proteomics (version 4.2; Waters Corporation) for chromatographic alignment and peak picking. Peptide identification was performed against the UniProt/SwissProt human proteome database (2022 release; 20,361 reviewed entries) with the following parameters: trypsin specificity with ≤1 missed cleavage; fixed modification: carbamidomethylation (Cys); variable modification: oxidation (Met); protein-level false discovery rate (FDR) < 1%. Protein quantification required ≥2 unique peptides per protein, ≥3 fragments per protein, and ≥2 fragments per peptide.

### 2.7. Data Preprocessing and Quality Control

Protein-level abundance values were exported from Progenesis QI. The ADH spike-in (P00330; 75 fmol per sample) served as an internal quality control standard and was excluded from downstream analyses. Zero abundance values were treated as missing (non-detected) and were not imputed.

Common serum contaminants were removed prior to statistical analysis, including hemoglobin subunits (HBA1, HBB, HBD, HBG2) and keratins (KRT1, KRT2, KRT9, KRT10, KRT77, KRT80). Proteins were retained if detected in ≥80% of samples in at least one group (PDAC or controls). After filtering, 407 proteins were retained for analysis.

### 2.8. Normalization and Transformation

Filtered protein abundances were log2-transformed to stabilize variance and reduce right-skewness. Global median normalization was performed by subtracting each sample’s median log2 intensity from all protein values within that sample, correcting for systematic differences in total protein loading.

### 2.9. Principal Component Analysis

Principal component analysis (PCA) was performed on log2-transformed, median-normalized abundance values. Protein features were mean-centered and scaled to unit variance. Only proteins with complete data across all samples were included in PCA.

### 2.10. Differential Abundance Analysis

Differential protein abundance between PDAC and control samples was assessed using Welch’s two-sample *t*-test applied to log_2_-transformed, median-normalized protein abundance values. Log_2_ fold change (log_2_FC) was calculated as the difference between the mean protein abundances in the PDAC and control groups (PDAC minus controls), and corresponding linear fold changes (FC) were derived accordingly. To account for multiple hypothesis testing, *p*-values were adjusted using the Benjamini–Hochberg false discovery rate (FDR) procedure. Proteins were considered significantly differentially abundant if they met all of the following criteria: nominal *p*-value < 0.05, FDR < 0.01, and an absolute fold change (|FC|) ≥ 1.5.

Two levels of false discovery rate (FDR) control were applied in this study. First, FDR control at the peptide and protein identification level was implemented during the mass spectrometry database search to limit incorrect peptide–spectrum matches. Second, multiple-testing correction during the statistical analysis of differential protein abundance was performed using the Benjamini–Hochberg procedure to control the false discovery rate across all quantified proteins.

### 2.11. Receiver Operating Characteristic Analysis

Receiver operating characteristic (ROC) analysis was performed to evaluate the diagnostic performance of individual proteins and multimarker panels. Analyses were conducted using log_2_-transformed protein abundance values that were median-normalized across samples to reduce inter-sample variation. PDAC samples were coded as positive cases (1) and control samples as negative cases (0).

For each quantified protein, the area under the ROC curve (AUC) was calculated to assess discriminatory ability. Sensitivity and specificity were determined at the classification threshold maximizing the Youden index (J = sensitivity + specificity − 1) in the discovery dataset; these values are reported as descriptive estimates rather than optimized diagnostic cutoffs. Proteins significantly increased in PDAC were subsequently ranked according to their AUC values in order to prioritize candidate biomarkers.

For multimarker panels, ROC analysis was applied to the predicted probabilities generated by logistic regression models. Diagnostic performance metrics included AUC, sensitivity, and specificity. Confidence intervals for AUC estimates were calculated using bootstrap resampling (1000 iterations). To improve transparency of diagnostic reporting, contingency table counts (true positives, false negatives, true negatives, and false positives) corresponding to the selected classification threshold were also reported.

### 2.12. Multimarker Diagnostic Modeling

Logistic regression models were constructed to evaluate the diagnostic performance of candidate protein combinations. Model discrimination was assessed using the area under the receiver operating characteristic curve (AUC). Internal validation was performed using repeated stratified five-fold cross-validation (100 repetitions) to estimate mean cross-validated AUC, and bootstrap resampling (1000 iterations) was applied to derive 95% confidence intervals using the percentile method.

Candidate selection strategy. Proteins were prioritized using a biologically informed approach combining three criteria: (i) significant differential abundance between PDAC and controls; (ii) individual diagnostic performance (AUC > 0.85); and (iii) functional enrichment for shared molecular pathways. This strategy was designed to identify biomarker panels with biological coherence rather than purely data-driven statistical associations. Because the primary objective was biomarker discovery rather than development of a finalized predictive classifier, candidate proteins were prioritized across the full discovery dataset prior to multimarker evaluation.

Methodological considerations regarding feature selection. We acknowledge that performing feature selection prior to cross-validation may introduce optimism bias, as the same data used for candidate prioritization were subsequently used for model evaluation. To mitigate information leakage, the final three-protein panel—comprising inter-alpha-trypsin inhibitor heavy chain 3 (ITIH3), coagulation factor XIII A chain (F13A1), and ferritin light chain (FTL)—was treated as a fixed, predefined feature set and evaluated using cross-validation without additional feature selection within validation folds. Nevertheless, the reported performance estimates should be interpreted as exploratory and potentially optimistic; independent validation in external cohorts will be required to determine the true diagnostic performance of the proposed biomarker panel.

Sensitivity analysis for demographic confounding. An exploratory sensitivity analysis assessed whether baseline demographic differences between groups influenced the observed biomarker performance. Three logistic regression models were compared: (i) demographic variables alone (age, sex, and diabetes status); (ii) biomarker variables alone (ITIH3, F13A1, FTL); and (iii) combined demographic and biomarker variables. Model discrimination was compared using AUC to determine whether the diagnostic signal was primarily attributable to the proteomic biomarkers or to demographic imbalances between cohorts.

Consequently, the reported performance metrics should be interpreted as internal validation estimates rather than unbiased estimates of true clinical performance.

### 2.13. Relationship Between the Protein Panel and CA19-9

To explore the relationship between the proteomic biomarker panel and the established pancreatic cancer marker carbohydrate antigen 19-9 (CA19-9), exploratory subgroup analyses were performed within the PDAC cohort for patients with available CA19-9 measurements. Patients were categorized according to the clinical CA19-9 cutoff of 34 U/mL. The classification performance of the three-protein panel was evaluated within CA19-9-defined subgroups to assess whether the proteomic biomarkers could detect PDAC cases with normal CA19-9 concentrations. Because CA19-9 measurements were not available for the control cohort, these analyses were interpreted as exploratory and descriptive.

### 2.14. Functional Enrichment Analysis

Functional enrichment was performed using g:Profiler (g:GOSt; https://biit.cs.ut.ee/gprofiler (accessed on 6 February 2026) for Homo sapiens against the default genome-wide background. Input consisted of proteins significantly increased in PDAC (*p* < 0.05, FDR < 0.01, |FC| ≥ 1.5) with AUC > 0.85. Gene Ontology (GO) categories and pathway databases (KEGG, Reactome) were queried, with emphasis on GO Cellular Component. Statistical significance was assessed using the hypergeometric test with g:SCS (set counts and sizes) correction; adjusted *p* < 0.05 was considered significant.

### 2.15. Clinicopathologic Association Analysis

Within the PDAC cohort, associations between top AUC-ranked proteins and clinicopathologic parameters (CA19-9, lymph node stage, T stage, tumor grade) were evaluated using Spearman rank correlation for ordinal/continuous variables and Mann–Whitney U tests for binary comparisons. BH correction was applied across the tested protein set within each clinical parameter.

### 2.16. Statistical Software

All data processing, statistical analyses, and visualizations were performed in Python (version 3.10) using the following packages: NumPy (version 1.24), Pandas (version 2.0), SciPy (version 1.11), scikit-learn (version 1.3), statsmodels (version 0.14), and Matplotlib (version 3.7). All statistical tests were two-sided unless otherwise specified.

### 2.17. Use of Generative Artificial Intelligence

Generative AI tools were used to assist with coding and statistical workflow implementation under direct author supervision. AI was not used to generate primary data, perform autonomous statistical interpretation, or draft scientific conclusions. All analytical procedures and outputs were independently reviewed and validated by the authors.

## 3. Results

### 3.1. Baseline Characteristics

The study cohort comprised 35 PDAC patients and 34 non-cancer controls. Demographic and clinical characteristics are summarized in Table 1.

### 3.2. Serum Proteome Characterization

Following data preprocessing and quality control filtering, 407 proteins were retained for downstream analyses, in line with previous publications [28]. These proteins exhibited robust quantitative coverage across both cohorts and constituted the basis for all subsequent statistical comparisons. The complete protein abundance matrix is provided in Supplementary Table S1.

### 3.3. Global Proteomic Patterns

**Figure 1:**
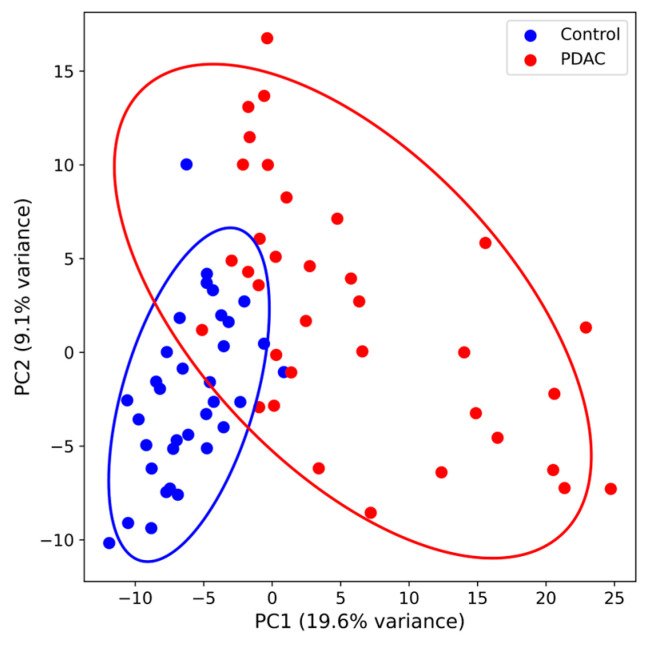
Principal Component Analysis of Serum Proteomic Profiles in PDAC and Control Cohorts. Figure 1 legend: Principal component analysis (PCA) of log2-transformed, globally median-normalized protein abundance values from DIA-based serum proteomics. Each point represents an individual sample (PDAC, n = 35, red; controls, n = 34, blue). PC1 explains 19.6% and PC2 explains 9.1% of total variance. Ellipses indicate 95% confidence regions. Partial separation along PC1 indicates measurable proteomic differences between groups, while greater dispersion within the PDAC cluster reflects inter-individual biological heterogeneity.

Principal component analysis revealed partial separation between PDAC and control samples along PC1, which explained 19.6% of total variance (Figure 1). Control samples formed a relatively compact cluster, whereas PDAC samples demonstrated greater dispersion, likely reflecting inter-individual variability in tumor biology and host response. No clustering indicative of technical bias was observed.

### 3.4. Differential Protein Abundance

**Figure 2:**
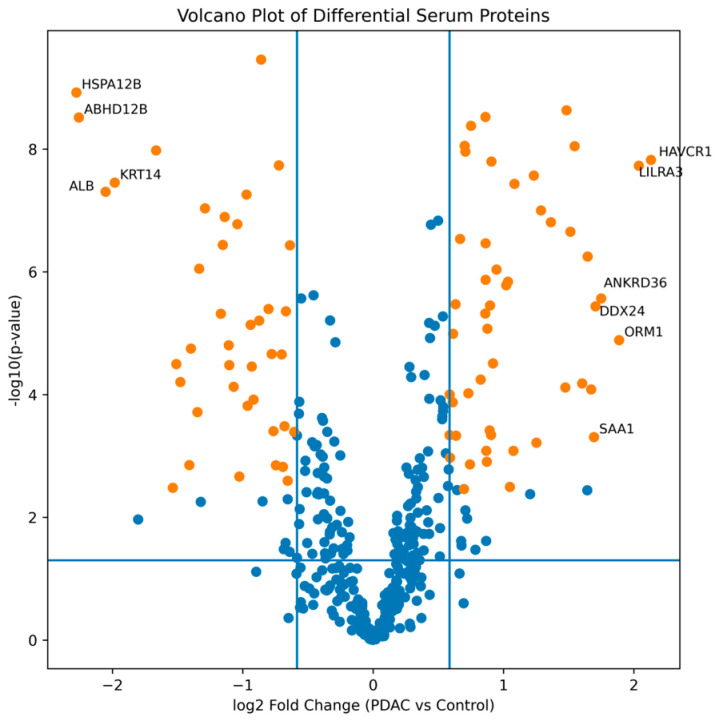
Differential Serum Protein Abundance in PDAC Compared with Controls. Figure 2 legend: Log2 fold change (PDAC vs. controls) is shown on the *x*-axis and −log10(*p*-value) from Welch’s *t*-test on the *y*-axis. Vertical lines indicate the fold change threshold (|log2FC| ≥ 0.58, corresponding to |FC| ≥ 1.5), and the horizontal line indicates the nominal significance threshold (*p* = 0.05). Proteins meeting the predefined criteria for differential abundance (*p* < 0.05, FDR < 0.01, and |FC| ≥ 1.5) are highlighted in orange: increased in PDAC on the right and decreased in PDAC on the left. Remaining proteins not meeting all criteria are shown in blue. Selected gene symbols are annotated for the most strongly altered proteins ranked by absolute fold change.

Ninety proteins met predefined significance criteria (*p* < 0.05, FDR < 0.01, |FC| ≥ 1.5), with 50 increased and 40 decreased in PDAC compared with controls. Proteins demonstrating the largest fold changes in the PDAC versus controls comparison included hepatitis A virus cellular receptor 1 (HAVCR1; FC 4.4) and leukocyte immunoglobulin-like receptor subfamily A member 3 (LILRA3; FC 4.1). The volcano plot in Figure 2 shows differential serum protein abundance in PDAC compared with controls, with proteins exhibiting the 10 largest fold changes labeled by their gene names. The complete list of differentially abundant proteins is provided in Supplementary Table S2.

### 3.5. Diagnostic Performance of Individual Proteins

Receiver operating characteristic (ROC) analysis identified several proteins with strong discriminatory capacity between PDAC and control serum samples (Table 2). Inter-alpha-trypsin inhibitor heavy chain H3 (ITIH3) demonstrated the highest diagnostic performance (AUC = 0.90), followed by coagulation factor XIII A chain (F13A1; AUC = 0.89) and ferritin light chain (FTL; AUC = 0.86). All top-ranked proteins satisfied the predefined differential abundance criteria, indicating strong concordance between statistical significance and diagnostic discrimination.

Serum CA19-9 levels were available only for the PDAC cohort and were therefore used exclusively for exploratory analyses rather than for ROC comparisons or multimarker modeling. Using the clinical cutoff of 34 U/mL, 10 of 29 PDAC patients (34%) had normal CA19-9 levels, whereas 19 patients (66%) showed elevated concentrations. Spearman correlation analysis between the three-protein panel score and CA19-9 levels demonstrated a weak, non-significant association (ρ = 0.29, *p* = 0.13), suggesting that the proteomic panel captures biological information partly independent of CA19-9. This observation indicates that the identified protein signature may provide complementary diagnostic information beyond CA19-9 alone. Supplementary Table S2 provides the detailed diagnostic performance results.

### 3.6. Functional Enrichment Analysis

**Figure 3:**
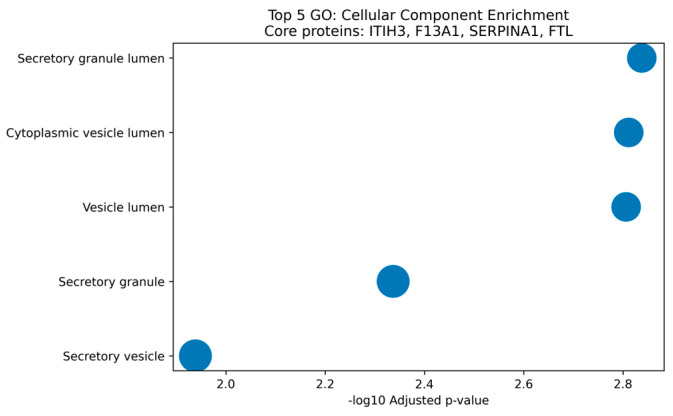
Functional Enrichment of High-Performing PDAC-Associated Serum Proteins. Figure 3 legend: Gene Ontology cellular component enrichment analysis of proteins significantly increased in PDAC with AUC > 0.85, performed using g:Profiler against the genome-wide Homo sapiens background. The *x*-axis represents -log10 adjusted *p*-value (g:SCS correction); dot size reflects gene ratio (overlapping genes/input genes). The top five enriched terms are shown. Secretory granule lumen (GO:0034774) was the most significantly enriched term (adjusted *p* = 0.00145), driven by inter-alpha-trypsin inhibitor heavy chain H3 (ITIH3), coagulation factor XIII A chain (F13A1), serpin family A member 1 (SERPINA1), and ferritin light chain (FTL). Complement and coagulation cascade pathway enrichment (adjusted *p* = 0.00096) was also observed.

Functional enrichment of proteins significantly increased in PDAC with AUC > 0.85 revealed significant overrepresentation of secretory granule lumen components (GO:0034774; adjusted *p* = 0.00145) and complement/coagulation cascades (adjusted *p* = 0.00096) (Figure 3). The secretory granule lumen enrichment was driven by ITIH3, F13A1, SERPINA1, and ferritin light chain (FTL), all significantly elevated in PDAC. The detailed results are shown in Supplementary Table S3.

### 3.7. Multimarker Diagnostic Biomarker Panel

Guided by functional enrichment analysis, biologically related candidate proteins were evaluated in combination using logistic regression models. Model performance was assessed using repeated stratified five-fold cross-validation (100 repetitions), and uncertainty of ROC estimates was evaluated using bootstrap resampling (1000 iterations).

**Figure 4:**
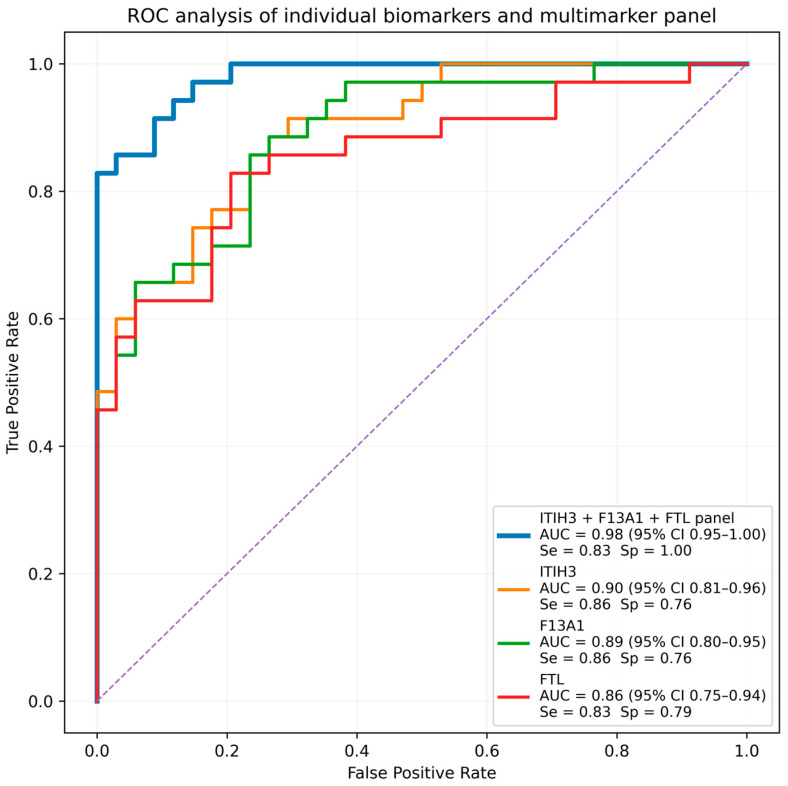
Diagnostic performance of individual biomarkers and the combined ITIH3–F13A1–FTL multimarker panel for discrimination of PDAC and control samples. Figure 4 legend: Receiver operating characteristic (ROC) curves are shown for individual proteins ITIH3 (AUC = 0.90, 95% CI 0.81–0.96; sensitivity 0.86, specificity 0.76), F13A1 (AUC = 0.89, 95% CI 0.80–0.95; sensitivity 0.86, specificity 0.76), and ferritin light chain (FTL; AUC = 0.86, 95% CI 0.75–0.94; sensitivity 0.83, specificity 0.79). The combined three-protein panel (ITIH3 + F13A1 + FTL), evaluated using logistic regression, demonstrated improved discrimination with AUC = 0.98 (95% CI 0.95–1.00). At the optimal classification threshold determined by the Youden index (0.635), the panel achieved sensitivity of 0.83 and specificity of 1.00. Protein abundance values were log_2_-transformed and globally median-normalized prior to analysis. Confidence intervals were estimated using bootstrap resampling. The dashed diagonal line represents the performance expected by random classification (AUC = 0.5).

Among the tested combinations, a three-protein panel comprising ITIH3, F13A1, and FTL demonstrated the strongest performance. Receiver operating characteristic (ROC) analysis of the combined panel fitted on the full discovery dataset yielded an area under the curve (AUC) of 0.98 (95% CI: 0.95–1.00) (Figure 4). Internal validation using repeated stratified cross-validation produced a mean AUC of 0.96, indicating stable model performance. Inclusion of additional candidate proteins did not improve model performance.

At the optimal classification threshold determined by the Youden index (0.635), the model correctly identified 29 of 35 PDAC patients and all 34 control subjects, corresponding to 6 false negatives and no false positives. This resulted in a sensitivity of 83% (95% CI: 66.4–93.4%) and specificity of 100% (95% CI: 89.7–100%). These results suggest that the enrichment-guided biomarker combination provides promising discrimination between PDAC and control samples within the discovery cohort.

### 3.8. Performance of the Proteomic Biomarker Panel Stratified by CA19-9 Status

CA19-9 measurements were available for 29 PDAC patients. Using the clinical cutoff of 34 U/mL, 10 patients had CA19-9 concentrations within the normal range and 19 had elevated CA19-9 levels.

The three-protein panel correctly classified 7 of 10 (70%) PDAC cases with normal CA19-9, indicating that the panel retains discriminatory capacity in patients who may not be detected using CA19-9 alone. Notably, approximately 5–10% of the population lacks Lewis antigen expression and cannot produce CA19-9, highlighting the clinical relevance of CA19-9-independent biomarkers. Among patients with elevated CA19-9, 17 of 19 (89%) were correctly classified by the protein panel.

These findings suggest partial complementarity between the proposed proteomic biomarker panel and CA19-9. However, because CA19-9 measurements were not available for the control cohort, formal comparative evaluation of diagnostic performance between CA19-9 and the proteomic panel could not be performed in the present dataset. Future studies including CA19-9 measurements in both PDAC cases and clinically relevant control populations will be necessary to determine whether combined biomarker strategies improve diagnostic accuracy.

### 3.9. Sensitivity Analysis Adjusting for Demographic Variables

To evaluate whether demographic differences between groups influenced the observed biomarker performance, an exploratory sensitivity analysis was performed using logistic regression models including age, sex, and diabetes status. A model based on demographic variables alone showed moderate discrimination between PDAC and controls (AUC = 0.71). In contrast, the three-protein biomarker panel (ITIH3, F13A1, and FTL) demonstrated substantially stronger discrimination (AUC = 0.93). A combined model including both biomarkers and demographic variables yielded a similar performance (AUC = 0.94), indicating that the diagnostic signal is primarily driven by the proteomic biomarkers rather than demographic differences between groups.

### 3.10. Clinicopathologic Associations

Within the PDAC cohort, associations between top-ranked proteins and clinicopathologic parameters were evaluated using Spearman correlation and Mann–Whitney U tests with BH correction.

**Figure 5:**
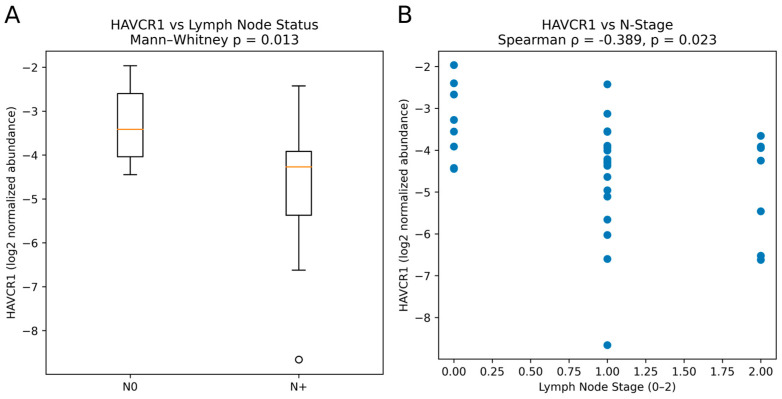
Association Between HAVCR1 Serum Abundance and Lymph Node Status in PDAC. Figure 5 legend: (**A**) Box plot showing HAVCR1 abundance (log2-transformed, globally median-normalized) in node-negative (N0, n = 12) and node-positive (N1/N2, n = 23) PDAC patients. Horizontal lines indicate median values; boxes indicate interquartile range; whiskers extend to 1.5× IQR. Mann–Whitney U test, *p* = 0.013. (**B**) Scatter plot of HAVCR1 abundance versus numeric lymph node stage (N0 = 0, N1 = 1, N2 = 2). Spearman rank correlation: ρ = −0.39, *p* = 0.023. Each point represents an individual patient. Higher HAVCR1 levels were observed in node-negative patients, suggesting an inverse association with regional disease dissemination.

Most prioritized biomarkers showed no significant association with lymph node status, T-stage, tumor grade, or CA19-9 levels. However, HAVCR1 demonstrated a significant inverse correlation with lymph node stage (Spearman ρ = −0.39, *p* = 0.023), with higher serum levels in node-negative (N0) compared with node-positive (N1/N2) patients (Mann–Whitney *p* = 0.013) (Figure 5). No other protein remained significant after multiple testing correction. Detailed results are presented in Supplementary Table S4.

## 4. Discussion

Using data-independent acquisition mass spectrometry, this discovery-phase study profiled the serum proteome of patients with resectable, non-metastatic pancreatic ductal adenocarcinoma to identify circulating biomarkers associated with disease presence. Differential abundance analysis and functional enrichment identified a three-protein signature comprising ITIH3, F13A1, and FTL, with enrichment for secretory granule lumen components and complement/coagulation pathways. The biological coherence of this panel suggests that resectable PDAC may be associated with coordinated systemic proteomic alterations—potentially reflecting tumor–stroma crosstalk and inflammatory reprogramming—that may be detectable in circulation before clinical progression. Global proteomic patterns

Principal component analysis demonstrated partial separation between PDAC and control samples, indicating that non-metastatic PDAC induces measurable systemic proteomic alterations. The heterogeneity observed within the PDAC group likely reflects inter-individual variability in tumor biology, inflammatory state, and host response—a pattern consistent with the known biological heterogeneity of PDAC [18,28]. The absence of clustering attributable to technical factors supports the robustness of the analytical pipeline. Differential abundance analysis revealed coordinated upregulation of proteins linked to secretion, inflammation, coagulation, and extracellular matrix remodeling rather than global proteome suppression. The enrichment of secretory granule lumen proteins (ITIH3, F13A1, SERPINA1, FTL) suggests that PDAC may alter regulated secretory pathways in hepatocytes, platelets, and immune cells—a hypothesis supported by recent spatial proteomic studies demonstrating that PDAC stroma actively modulates systemic protein secretion through paracrine signaling [20,29,30,31,32]. This mechanistic framework distinguishes our findings from non-specific acute-phase responses and suggests that the identified proteins reflect tumor-specific biology rather than generalized inflammation.

### 4.1. Biological Significance of Individual Biomarkers

ITIH3 achieved the highest individual AUC (0.90), with the observed 1.8-fold increase aligning closely with prior independent studies reporting similar elevations in PDAC compared with both healthy controls and chronic pancreatitis [24,29]. Notably, a recent study using genetically engineered mouse models and human samples confirmed that ITIH3 levels were significantly elevated across PanIN-abundant mice, KPC mice, and early-stage PDAC patients, supporting its potential as a biomarker for precancerous lesions and early-stage disease [33].Mechanistically, ITIH3 participates in extracellular matrix stabilization and hyaluronan binding, suggesting it reflects microenvironmental remodeling rather than non-specific inflammation [34]

F13A1 reinforces the recognized link between PDAC and hypercoagulability. Pancreatic cancer is characterized by high tumoral expression of tissue factor, activation of leukocytes with release of neutrophil extracellular traps, and dissemination of tumor-derived microvesicles that promote hypercoagulability [35].Coagulation-related proteins have been identified as enriched in extracellular vesicles derived from pancreatic cancer cells, supporting a mechanistic role for coagulation pathway activation in tumor progression [30]. The complement and coagulation cascade enrichment observed in our functional analysis aligns with this biology and with emerging evidence linking thromboinflammation to PDAC pathogenesis [11], including recent single-cell sequencing studies demonstrating high expression of coagulation factors in cancer-associated fibroblasts [36,37].

FTL, representing iron metabolism dysregulation, is supported by large consortium-based genetic analyses implicating iron regulatory pathways in PDAC susceptibility [31]. Dysregulated iron homeostasis has been increasingly recognized as a hallmark of pancreatic cancer, with ferritin serving as both a marker of altered iron metabolism and a potential contributor to tumor progression through oxidative stress mechanisms [38] SERPINA1 overexpression has been documented in PDAC tissue and associated with adverse prognosis, with circulating elevation supporting biomarker potential [32,39,40].

### 4.2. Multimarker Panel Performance and Internal Validation

Our three-protein panel (AUC 0.98, cross-validated mean AUC 0.96) achieves internal validation performance comparable to recently validated multimarker panels. A sensitivity analysis incorporating age, sex, and diabetes status demonstrated that demographic variables alone provided only moderate discrimination (AUC = 0.71), whereas the proteomic biomarker panel retained strong diagnostic performance (AUC = 0.93), suggesting that the observed signal is primarily driven by the circulating protein markers rather than baseline demographic differences.

An exploratory analysis evaluated the relationship between the proposed proteomic biomarker panel and CA19-9. Among PDAC patients with available CA19-9 measurements, the three-protein panel correctly classified 7 of 10 cases (70%) with CA19-9 concentrations within the normal range. This observation suggests that the proteomic panel may detect a subset of PDAC cases that would not be identified using CA19-9 alone, supporting potential complementary value. However, because CA19-9 measurements were not available for the control cohort, direct comparison of diagnostic performance was not possible. Future studies incorporating parallel measurement of CA19-9 and proteomic markers in both PDAC cases and clinically relevant control populations will be required to determine whether combined biomarker strategies improve diagnostic accuracy.

### 4.3. Comparison with Existing Multimarker Panels

Multiple independent studies support the superiority of multimarker panels over CA19-9 alone for PDAC detection. The PancreaSure panel (TIMP1, ICAM1, CTSD, THBS1, CA19-9) achieved 0.78 sensitivity and 0.93 specificity in an independent cohort of 1066 samples, significantly outperforming CA19-9 alone [41]. The CA19-9/THBS2/ANPEP/PIGR combination achieved 0.88 sensitivity for stage I/II disease at 0.95 specificity in multi-center validation [42]. A combined panel of CA19-9, TIMP1, and methylated cfDNA achieved AUC 0.92 for early-stage PDAC in independent validation [14]. Recent machine learning approaches combining CA19-9, GDF15, and suPAR achieved AUC values of 0.976–0.992 for early-stage PDAC detection [43].

Multiple independent studies support the superiority of multimarker panels over CA19-9 alone. Kim et al. demonstrated improved diagnostic performance using a six-marker proteomic panel achieving high diagnostic accuracy in training and validation cohorts [23]. Tonack et al. incorporated ITIH3 into a combinatorial signature improving discrimination between PDAC and benign disease [24,]. Capello et al. validated a TIMP-1/LRG1/CA19-9 panel across independent cohorts [25]. Additional glycoprotein and MS-based panels have similarly demonstrated improved accuracy [26,27].

However, direct comparison with our findings is limited by critical differences in study design. Our cohort lacked chronic pancreatitis controls, which typically reduce biomarker performance by 15–25% compared with healthy control comparisons [44]. A recent comprehensive meta-analysis demonstrated that novel protein biomarkers, while showing moderate diagnostic accuracy, did not consistently outperform CA19-9 in differentiating PDAC from benign disease and showed limited added clinical value when combined with CA19-9 [45]. Meta-analyses demonstrate that CA19-9 achieves only moderate discrimination between PDAC and chronic pancreatitis (pooled sensitivity 0.70, specificity 0.86), with performance further reduced in early-stage disease [10]. The absence of CA19-9 measurement in our control group precludes assessment of additive value over the current standard. Although CA19-9 remains the only approved PDAC biomarker, its limitations in early-stage disease and Lewis antigen–negative individuals are well recognized [46]. Our findings should therefore be interpreted as complementary rather than competitive with CA19-9; future studies incorporating parallel CA19-9 assessment across all groups will be required to determine additive or synergistic diagnostic value.

### 4.4. Clinicopathologic Associations

Clinicopathologic analyses indicated that most prioritized biomarkers were independent of lymph node status, suggesting that they primarily reflect systemic disease presence rather than regional dissemination. HAVCR1 demonstrated only nominal association with nodal stage. While HAVCR1 tissue overexpression has been linked to immune modulation and advanced PDAC, circulating detection appears less consistent, aligning with our observations [47,48,49].

### 4.5. Interpretation of Diagnostic Performance Estimates

Although the multimarker panel demonstrated strong discriminatory performance in the present analysis (AUC = 0.98), these estimates were obtained within a discovery cohort and should therefore be interpreted cautiously. Cross-validation indicated stable model performance, suggesting that the observed diagnostic signal is not driven by a small subset of samples. However, because feature selection was performed prior to cross-validation, the reported performance estimates may be optimistic. Independent validation in larger, prospectively collected cohorts will be required to determine the true clinical performance and generalizability of the ITIH3–F13A1–FTL biomarker panel.

### 4.6. Limitations

This study has several limitations inherent to discovery-phase biomarker research. First, the control cohort did not include clinically relevant comparator conditions such as chronic pancreatitis or benign biliary obstruction—populations that represent the true diagnostic differential for PDAC and in which biomarker performance typically decreases by 15–25%. The reported diagnostic metrics should therefore be interpreted as exploratory signals rather than estimates of real-world clinical accuracy. Second, the modest cohort size, determined by feasibility constraints of high-resolution mass spectrometry workflows and strict clinical inclusion criteria, may increase the risk of effect-size inflation and overfitting. Third, baseline demographic differences between groups—particularly age (SMD = 0.71) and sex (SMD = 0.45)—may introduce residual confounding, although the biomarker panel demonstrated substantially stronger discrimination than demographic variables alone. Fourth, candidate proteins were prioritized across the full dataset prior to model evaluation, which may introduce optimism bias despite cross-validation and bootstrap resampling. However, the multimarker panel was selected primarily on the basis of biological coherence identified through functional enrichment analysis rather than purely data-driven optimization, which may reduce the likelihood of overfitting. Nevertheless, the diagnostic performance of the ITIH3–F13A1–FTL panel should be interpreted as exploratory and will require confirmation in independent validation cohorts. Fifth, CA19-9 measurements were available only for the PDAC cohort, precluding direct head-to-head diagnostic comparison with the proteomic panel. Finally, the cross-sectional design precludes assessment of temporal biomarker dynamics or pre-diagnostic detection capability.

### 4.7. Strengths

Despite these limitations, the study has notable strengths. The exclusive focus on resectable, non-metastatic PDAC minimizes confounding from advanced disease burden—a common limitation in prior proteomic studies that included mixed-stage populations [22,23,24,25,26]. The reproducible DIA-based workflow, stringent statistical thresholds (FDR < 0.01, |FC| ≥ 1.5), and biologically informed candidate prioritization through functional enrichment analysis enhance the robustness and reproducibility of the findings. Internal validation using repeated cross-validation and bootstrap resampling supported the stability of the three-protein panel within the discovery cohort.

### 4.8. Future Directions

Translational validation of the ITIH3–F13A1–FTL panel will require several steps. First, external validation in larger, independent multi-center cohorts—including chronic pancreatitis and benign pancreatic disease controls—will be essential to establish diagnostic specificity in clinically relevant populations. Second, parallel assessment alongside CA19-9 will be necessary to determine whether the panel provides additive or complementary diagnostic value, particularly in Lewis antigen–negative individuals who cannot produce CA19-9. Third, development of targeted clinical assays, such as ELISA-based platforms or targeted mass spectrometry (e.g., selected reaction monitoring), will be required for practical implementation and standardization across clinical laboratories. Fourth, prospective longitudinal studies in high-risk populations will be needed to assess whether these biomarkers can detect PDAC prior to clinical diagnosis and contribute to improved patient outcomes. Finally, integration with emerging liquid biopsy approaches—including circulating tumor DNA and extracellular vesicle-based signatures—may further enhance diagnostic performance [50].

## 5. Conclusions

This discovery-phase proteomic study identified a three-protein serum signature comprising ITIH3, F13A1, and FTL that shows promising discriminatory capacity between patients with resectable pancreatic ductal adenocarcinoma and non-cancer controls within the discovery cohort. Functional enrichment analysis revealed biological coherence, with significant overrepresentation of secretory granule lumen components and complement/coagulation pathways—processes mechanistically linked to PDAC tumor–stroma interactions and systemic inflammatory reprogramming.

The exclusive focus on resectable, non-metastatic disease addresses a critical gap in PDAC biomarker research, as most prior studies have included advanced-stage patients whose circulating profiles may reflect tumor burden rather than early disease biology. The observation that the proteomic panel correctly classified 70% of PDAC cases with normal CA19-9 levels suggests potential complementary diagnostic value, although this requires formal validation.

The reported diagnostic performance (AUC 0.98, cross-validated AUC 0.96) should be interpreted as exploratory given the case–control design, absence of clinically relevant comparator populations such as chronic pancreatitis, and potential for optimism bias inherent to discovery-phase analyses. Independent external validation in larger, multi-center cohorts—including benign pancreatic disease controls and parallel CA19-9 assessment—will be essential to determine the true clinical applicability of this biomarker panel.

Given the aggressive biology and silent progression of PDAC, discrimination at a resectable stage remains the most critical determinant of long-term survival. The integration of reliable circulating biomarkers with established clinical and imaging modalities may ultimately improve identification of patients with early-stage disease, enabling timely curative-intent resection and potentially transforming outcomes in this challenging malignancy.

## Figures and Tables

**Table 1:** Baseline Demographic and Clinical Characteristics of the Study Cohort.

Characteristic	Controls (n = 34)	PDAC (n = 35)	SMD
Age (years), mean ± SD	55.2 ± 14.2	63.9 ± 9.9	0.71
Male sex, n (%)	24 (70.6%)	17 (48.6%)	0.45
Any comorbidity, n (%)	16 (47.1%)	26 (74.3%)	0.56
Chronic pancreatitis, n (%)	0 (0.0%)	5 (14.3%)	0.55
Obesity (BMI > 30), n (%)	4 (11.8%)	1 (2.9%)	0.35
Hypertension, n (%)	13 (38.2%)	21 (60.0%)	0.44
Type 2 diabetes, n (%)	2 (5.9%)	15 (42.9%)	0.86
CA19-9 (U/mL), median (IQR)	–	68.6 (25.3–312.0)	-
CEA (ng/mL), median (IQR)	–	2.5 (1.8–4.7)	-

Footnote: Data are presented as mean ± standard deviation (SD) for normally distributed continuous variables, median with interquartile range (IQR) for non-normally distributed continuous variables, or n (%) for categorical variables. Standardized mean differences (SMD) were calculated to quantify baseline imbalances; SMD > 0.20 indicates potentially meaningful differences. CA19-9 and CEA were measured only in the PDAC cohort. Control subjects had no known hepato-bilio-pancreatic disease. Abbreviations: BMI, body mass index; CA19-9, carbohydrate antigen 19-9; CEA, carcinoembryonic antigen; PDAC, pancreatic ductal adenocarcinoma; SMD, standardized mean difference.

**Table 2:** Diagnostic Performance of Top 15 Serum Proteins Increased in PDAC.

	Protein	Gene	FC	AUC	Se	Sp	95%CI
1	Inter-alpha-trypsin inhibitor heavy chain H3	ITIH3	1.8	0.90	0.86	0.76	0.81–0.96
2	Coagulation factor XIII A chain	F13A1	1.7	0.89	0.86	0.76	0.80–0.95
3	Neutrophil defensin 1	DEFA1	2.9	0.88	0.71	0.97	0.79–0.96
4	Mannan-binding lectin serine protease 2	MASP2	1.9	0.88	0.80	0.76	0.79–0.95
5	Leukocyte immunoglobulin-like receptor subfamily A member 3	LILRA3	4.1	0.88	0.66	0.97	0.79–0.95
6	Long-chain fatty acid-CoA ligase 5	ACSL5	1.6	0.87	0.71	0.97	0.79–0.95
7	Coiled-coil domain-containing protein 141	CCDC141	2.1	0.87	0.91	0.68	0.79–0.95
8	Hepatitis A virus cellular receptor 1	HAVCR1	4.4	0.87	0.74	0.94	0.78–0.95
9	Cadherin-related family member 2	CDHR2	2.8	0.87	0.69	0.97	0.79–0.94
10	Thyroid receptor-interacting protein 11	TRIP11	1.6	0.87	0.83	0.82	0.76–0.94
11	Alpha-1-antitrypsin	SERPINA1	2.3	0.86	0.80	0.85	0.75–0.94
12	Leucine-rich repeat and IQ domain-containing protein 1	LRRIQ1	2.4	0.86	0.77	0.82	0.76–0.94
13	Ferritin light chain	FTL	2.0	0.86	0.83	0.79	0.75–0.94
14	Plastin-2	LCP1	1.6	0.85	0.80	0.76	0.76–0.93
15	Thrombospondin-4	THBS4	2.6	0.85	0.86	0.76	0.71–0.93

Footnote: Proteins significantly increased in PDAC (*p* < 0.05, FDR < 0.01, |FC| ≥ 1.5) ranked by area under the receiver operating characteristic curve (AUC). ROC analysis was performed on log2-transformed, globally median-normalized abundance values comparing PDAC (n = 35) with controls (n = 34). Fold change (FC) represents PDAC relative to controls. Sensitivity (Se) and specificity (Sp) were calculated at the optimal classification threshold determined by the Youden index. The area under the receiver operating characteristic curve (AUC) and corresponding 95% confidence intervals (95% CI) were estimated using bootstrap resampling with 1000 iterations. Abbreviations: AUC, area under the curve; FC, fold change; FDR, false discovery rate; PDAC, pancreatic ductal adenocarcinoma; Se, sensitivity; Sp, specificity; 95% CI, 95% confidence intervals.

## Data Availability

The authors will provide the raw data supporting this article’s conclusions upon request, without restriction.

## Supplementary Materials

The following supporting information can be downloaded at: https://www.mdpi.com/article/10.3390/medicina62030605/s1, Supplementary Methods: Detailed Mass Spectrometry Instrument Parameters; Supplementary Table S1: Complete Protein Abundance Matrix for All Quantified Serum Proteins; Supplementary Table S2: Differential Abundance and Diagnostic Performance Analysis; Supplementary Table S3: Functional Enrichment Analysis Results (gProfiler Output); Supplementary Table S4: Clinicopathologic Association Analysis for Top-Ranked Proteins.

## Abbreviations

The following abbreviations are used in this manuscript:

AUC	Area under the receiver operating characteristic curve
BH	Benjamini–Hochberg
BMI	Body mass index
CA19-9	Carbohydrate antigen 19-9
CEA	Carcinoembryonic antigen
CI	Confidence interval
DIA	Data-independent acquisition
F13A1	Coagulation factor XIII A chain
FC	Fold change
FDR	False discovery rate
FTL	Ferritin light chain
GO	Gene Ontology
HAVCR1	Hepatitis A virus cellular receptor 1
HDMSE	High-definition mass spectrometry with elevated energy
IQR	Interquartile range
ITIH3	Inter-alpha-trypsin inhibitor heavy chain H3
KEGG	Kyoto Encyclopedia of Genes and Genomes
MASP2	Mannan-binding lectin serine protease 2
PCA	Principal component analysis
PDAC	Pancreatic ductal adenocarcinoma
ROC	Receiver operating characteristic
SD	Standard deviation
SERPINA1	Serpin family A member 1
