# Supplementary material for: Discovery of a Secretory Granule Lumen-Enriched Serum Protein Signature in Resectable Pancreatic Ductal Adenocarcinoma
Source: Medicina (Kaunas). 2026 Mar 23;62(3):605. doi: 10.3390/medicina62030605 (PMC13027620; doi:10.3390/medicina62030605)
Supplement: Supplementary file 1 [file medicina-62-00605-s001.zip › Supplementary Methods_Detailed Mass Spectrometry Instrument Parameters.pdf]

Supplementary Methods. Detailed Mass Spectrometry Instrument Parameters.

*Description of experiment settings for LC-$MS^E$ analysis and identification of proteins*

| | |
|---|---|
| **LC-Parameters** | |
| LC gradient<br>A: 0.1 % formic acid in water<br>B: 0.1 % formic acid in acetonitrile | 0min-5% B-5m-5%-95m-40%-100m-85%-105m-85%-106m-5%-120m-5% |
| Flow rate | 0.400µL/min |
| Column temperature | 55°C |
| **$MS^E$-Parameters** | |
| Lock spray | GluFib precursor 785.8426 m/z |
| Acquisition times | 10-110min |
| Acquisition mode | positive, resolution |
| Mass range | 50-2000Da |
| Capillary voltage | 3kV |
| Sampling cone | 40V |
| Source offset | 80V |
| Source temperature | 80°C |
| Nebuliser gas flow | 6.5 bar |
| Trap collision energy | 4V |
| Transfer collision energy | 2V |
| **Protein search parameter** | |
| Name of peak list-generating software and release version (number or date) | Progenesis QI v4.2 (Waters Corporation) |
| Name of the search engine and release version (number or date) | MSE built in search engine of Progenesis QI v4.2 (Waters Corporation) |
| Name of database searched and release version/date | Swissprot database release 2024 limited to human entries |
| Enzyme specificity considered | Fully tryptic |
| # of missed cleavages permitted | 1 |
| Fixed modification(s) (including residue specificity) | carbamidomethylation of cysteine |
| Variable modification(s) (including residue specificity) | oxidation of methionine |
| Fragment ions per protein | 1 |
| Fragment ions per peptide | 3 |
| Threshold score for accepting protein identification | ≥2 significant peptides |
| Threshold score/E-value for accepting individual MS/MS Spectra | False discovery rate at peptide level <1% |
| software/method used to evaluate site assignment | No PTM reported |
